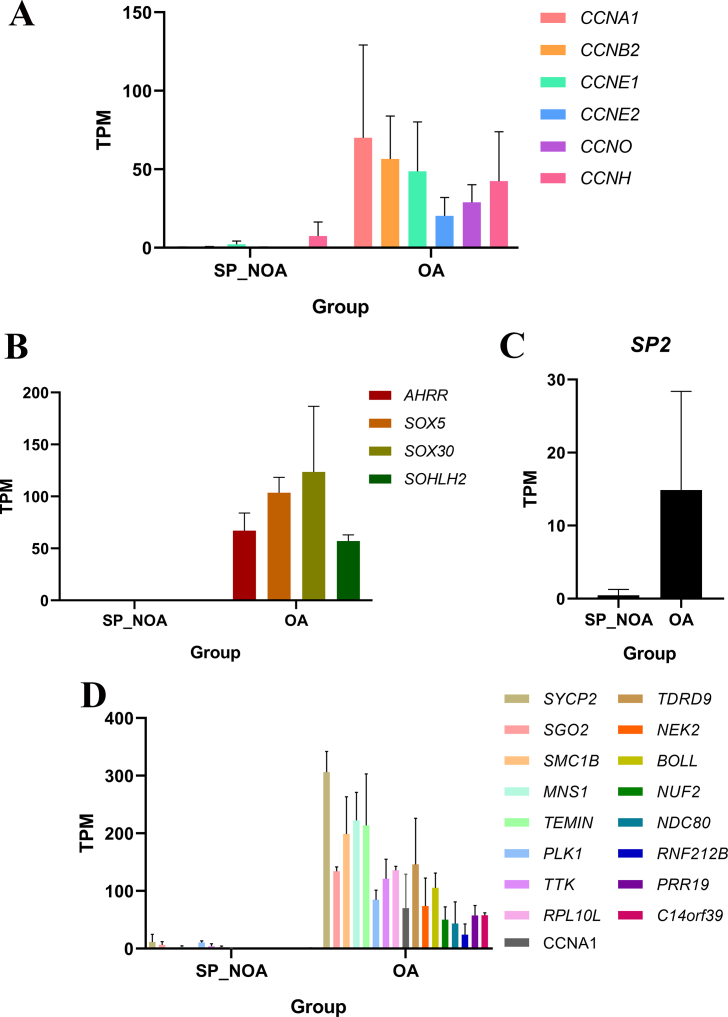# *In situ* transcriptomic analysis of spermatocytes in non-obstructive azoospermia reveals senescence-like states in arrested spermatocytes

**DOI:** 10.1016/j.gendis.2024.101205

**Published:** 2024-01-03

**Authors:** Zhongqin Wu, Xiaolong Wu, Zeqian Xu, Yixin Liu, Ziyi Zhu, Xinhui Li, Daniel M. Czajkowsky, Fei Sun, Yan Guo, Zhifeng Shao

**Affiliations:** a State Key Laboratory of Systems Medicine for Cancer, School of Biomedical Engineering and Bio-ID Center, Shanghai Jiao Tong University, Shanghai 200240, China; b Department of Urology and Andrology, Sir Run Run Shaw Hospital, Zhejiang University School of Medicine, Hangzhou, Zhejiang 310016, China

Germ cell arrest is one kind of important disease in non-obstructive azoospermia (NOA) including spermatogonia arrest, spermatocyte arrest, and round spermatid arrest.^1^ There is an urgent need to explore the molecular mechanisms underlying germ cell arrest, which are essentially unknown and could provide not only a promising approach for therapy but also novel possibilities for developing male contraceptives.

Since testicular tissue is highly heterogeneous and composed of various cell types, examining the *in situ* transcriptomes of specific cell types of the maturation process is essential. In this communication, we analyzed nine testicular biopsies from nine patients diagnosed with azoospermia (Table S1): three patients with NOA arrested at the spermatocyte stage (SP_NOA), three patients with NOA arrested at the round spermatid stage (rST_NOA), and three patients with obstructive azoospermia (OA). NOA patients with genetic problems, including abnormal karyotypes and Y chromosome micro-deletion, and other influencing factors were all excluded in this study. We used laser capture microdissection to dissect approximately 150 spermatocytes (including primary and secondary spermatocytes) from formalin-fixed paraffin-embedded testicular biopsies shown in Figure 1A. Sequencing libraries were constructed using the Smart-3SEQ method.^2^ On average, approximately 18,000 expressed genes, including more than 1000 transcription factors (TPM > 1), were detected for each sample (Table S2), indicating that these transcriptomes are of excellent quality for detailed analyses.

**Figure 1:**
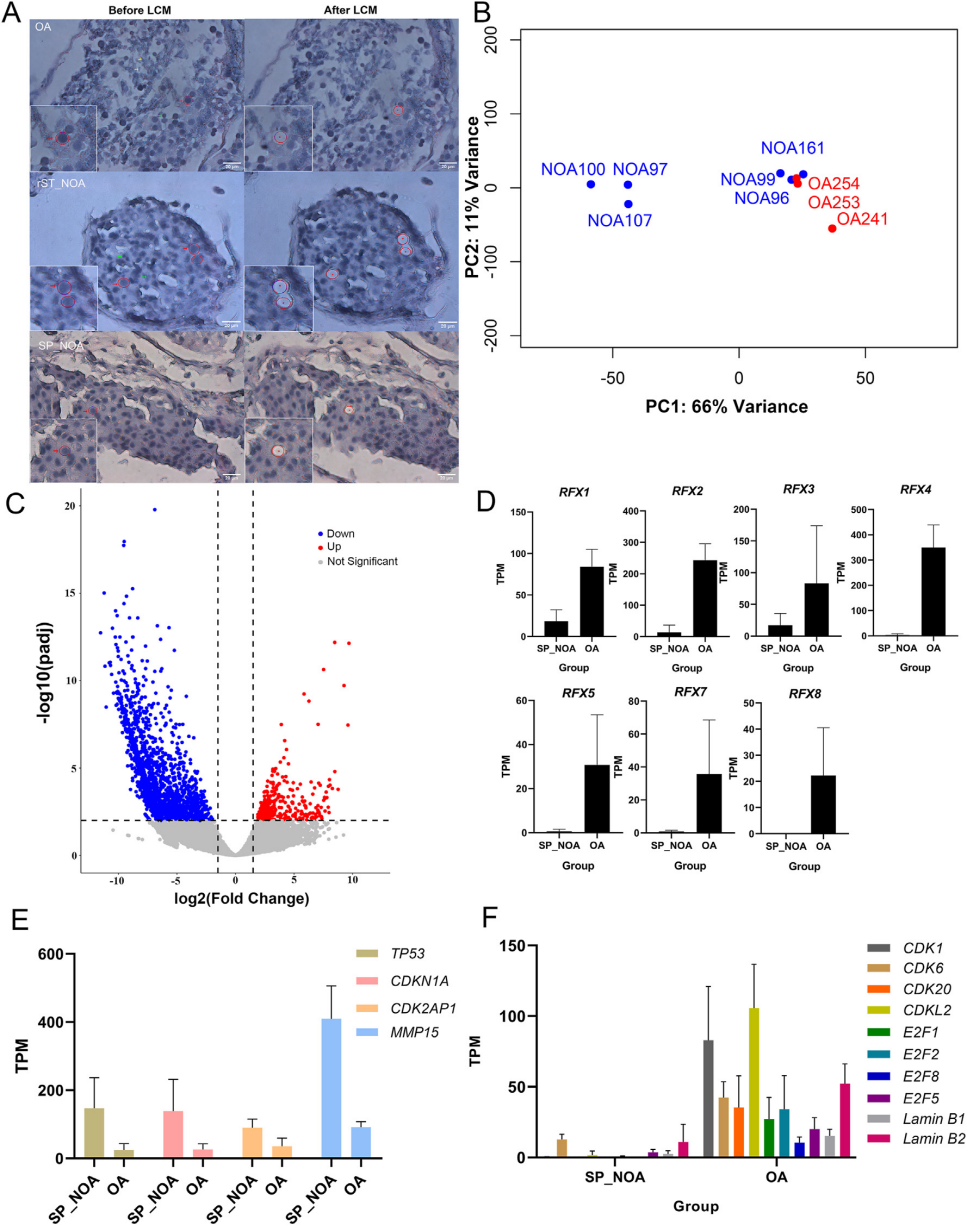
Comparison of *in situ* transcriptome of spermatocytes between two types of non-obstructive azoospermia (NOA) and control samples in human testicular FFPE biopsies. **(A)** Utilizing laser capture microdissection, spermatocytes were isolated from testicular FFPE sections following hematoxylin staining with single-cell resolution, including **(a)** spermatocytes isolated from obstructive azoospermia samples (OA, control sample with normal spermatogenesis), **(b)** spermatocytes isolated from rST_NOA samples (NOA arrested at the round spermatid stage), and **(c)** spermatocytes isolated from SP_NOA samples (NOA arrested at the spermatocyte stage). Red arrow: spermatocyte; green arrow: round spermatid; yellow arrow: elongated spermatid. **(B)** Principal component analysis revealed two distinct clusters: one formed by the SP_NOA group and the other comprising rST_NOA and OA groups. This clustering indicated a remarkable similarity in the transcriptome of spermatocytes between the OA and rST_NOA groups while underscoring distinguishable differences from the SP_NOA group. **(C)** The volcano plot showing significant variations in spermatocyte profiles between the SP_NOA and control groups. The threshold was set |log_2_ (fold change)| > 1.5 and adjusted *P* value < 0.01 for markedly altered expression. The red dots showed up-regulated genes in the SP_NOA group, and the blue dots showed down-regulated genes in the SP_NOA group. **(D)** The gene expression levels (quantified in TPM values) of seven transcription factors (RFX1-5, RFX7, RFX8) were greatly depressed in the SP_NOA group. **(E, F)** The gene expression status (quantified in TPM values) of senescence-related genes in the SP_NOA group remarkably resembled those observed in senescent cells.

Principal component analysis of the transcriptomes showed that the samples were segregated into two groups (Fig. 1B): the SP_NOA samples formed one cluster while the rST_NOA and OA formed another cluster. This indicates that, at the transcriptomic level, the samples of the round-spermatid stage are quite similar to the normal control, whereas those arrested at the spermatocyte stage are significantly different. We then examined the differentially expressed genes between different samples using DESeq2 (adjusted *p* value < 0.01, |log_2_ (fold change)| > 1.5). Consistent with the principal component analysis results, there were no significantly differentially expressed genes between the rST_NOA samples and OA samples. By contrast, we identified 2059 down-regulated genes and 301 up-regulated genes in the SP_NOA samples when compared with OA samples (Fig. 1C). Interestingly, of the down-regulated genes, 1739 genes were completely undetectable in the SP_NOA patients. Gene ontology analysis (Gene Annotation File 2.2) indicated that these completely suppressed genes are enriched in pathways of male gamete generation, cilium movement, meiotic cell cycle, and nuclear chromosome segregation (Fig. S1A), which is generally consistent with the arrested nature of these spermatocytes. A noteworthy example is the cyclin family genes (Fig. S2A). *CCNA1*, *CCNB2*, *CCNE1*, and *CCNE2* have been reported to associate with NOA patients and the lack of *CCNE2* and *CCNO* expression has not been described before.

The altered expression of regulators is of particular interest, owing to the critical importance of these factors in cellular development and differentiation. We found that 66 transcription factors lost their expression completely in the SP_NOA samples (Table S3), and most of them were associated with the meiotic cell cycle and spermatogenesis. The function of some master regulators has been validated in a mouse model. *E2F1*-null mice develop cryptorchidism, characterized by severe gubernacular defects and progressive loss of germ cells, ultimately leading to infertility.^3^ The absence of BNC1 significantly affected the reproductive capacity of both juvenile and adult male mice.^4^ These changes are also in line with the arrested nature of differentiation in these spermatocytes.

Although the inter-relationship of these transcription factors and their specific functions in the etiology of this disease requires detailed examination, the *RFX* family may warrant particular attention. We found that all *RFX* genes were substantially down-regulated in SP_NOA samples (Fig. 1D) except *RFX6* which was not expressed in either control or SP_NOA patients. In fact, *RFX4*, *5*, *7*, and *8* were not expressed at all in SP_NOA samples. Although the upstream regulators of *RFX* genes are not well understood, *SP2* is known to regulate *RFX5*, and this transcription factor was also not expressed in the SP_NOA patients (but expressed in OA) (Fig. S2C). Further analysis of the promoter regions of differentially expressed genes for *RFX* motif enrichment identified 17 genes involved in meiosis that were dramatically down-regulated in SP_NOA patients (Fig. S2). The marked down-regulation of these genes provides a potential mechanism for spermatocyte arrest that may be further examined in detail.

We note that the up-regulated differentially expressed genes in SP_NOA patients, though fewer in number, are perhaps more intriguing. Gene ontology analysis of the activated genes (only expressed in SP-NOA but not in control) revealed pathways associated with immune responses (Fig. S1B). Since activated immune pathways and cell-cycle arrest are two major characteristics of senescent cells, which is a complex cellular stress response state that limits cell proliferation, we further examined the expression status of known senescence-related genes^5^ and found intriguing similarities. The most salient feature of cellular senescence is proliferative arrest mediated by the activation of tumor suppressors and downstream effectors, which inhibit cyclin-dependent kinases (CDKs) and transcriptional activators (namely, the E2F family) that drive the cell cycle.^5^ *TP53*, a tumor suppressor, and its effectors *CDKN1A* and *CDK2AP1* were found substantially elevated in arrested spermatocytes, while *CDK1*, *CDK6*, *CDK20*, and *CDKL2* were all significantly down-regulated (Fig. 1E, F). As for the *E2F* family, *E2F1*, *E2F2*, and *E2F8* completely lost their expression in arrested spermatocytes while *E2F5* was down-regulated (Fig. 1F). Matrix metalloproteases 15 (*MMP15*), a known up-regulated gene in senescent cells, was also up-regulated in arrested spermatocytes (Fig. 1E). Moreover, arrested spermatocytes also lost their lamin B1/B2 expression, similar to senescent cells (Fig. 1F).

In summary, recognizing the potential introduction of nonnegligible gene expression bias associated with single-cell dissociation conditions in single-cell transcriptome analysis, we opted to characterize the *in situ* transcriptomes of spermatocytes isolated from NOA patients. We found that only the arrested spermatocytes differ substantially in expression from their normal counterparts but the spermatocytes from round spermatid arrested patients do not, implying that mechanisms of NOA are arresting stage-specific. With the arrested spermatocytes, we discovered a large number of substantially down-regulated genes, providing a significant set of genes and pathways for further analysis. We also found that the up-regulated genes in the arrested spermatocytes were enriched in pathways associated with immune responses, suggesting that chronic inflammation might play an important role in these patients. More intriguing, these arrested spermatocytes also dysregulated many senescence-related genes, indicating that these cells also adopt characteristics of this phenotype. It is worth noting that these dysregulated genes need further validation with quantitative PCR when fresh frozen testis tissue from patients who are not genetically predisposed to this disease is available. If senescence-like states are the common mechanism for NOA, further investigation on specific arrested cells, such as round spermatid in rST_NOA samples, is needed. Therefore, whether anti-senescence therapies, such as senolytics, could have beneficial effects is an interesting aspect to examine. Moreover, the large number of down-regulated genes found in the arrested spermatocytes also raises the possibility of targeting these genes for developing novel male contraceptives, an opportunity worth further investigation.

## Ethics declaration

FFPE blocks of human testicular tissue in this study were provided by Dr. Xiaolong Wu, Zhejiang University School of Medicine, which was approved by the Human Ethics Committee, Sir Run Shaw Hospital, Zhejiang University School of Medicine, Hangzhou, Zhejiang, China, with the approval number 20210825–30.

## Conflict of interests

The authors declare no conflict of interests.

## Funding

This work was supported by the 10.13039/501100001809National Natural Science Foundation of China (No. 81972909, 31971151), the 10.13039/501100012166National Key Research and Development Program of China (No. 2021YFC2700200), the Bio-ID Center at SJTU, and the K.C. Wong Education Foundation (H.K.).

## Data availability

The raw data that support the findings of this study are openly available in NCBI's Gene Expression Omnibus and are accessible through https://www.ncbi.nlm.nih.gov/geo/query/acc.cgi?acc=GSE238078, GEO Series accession number [GSE238078].

## Figures and Tables

**figs1:**
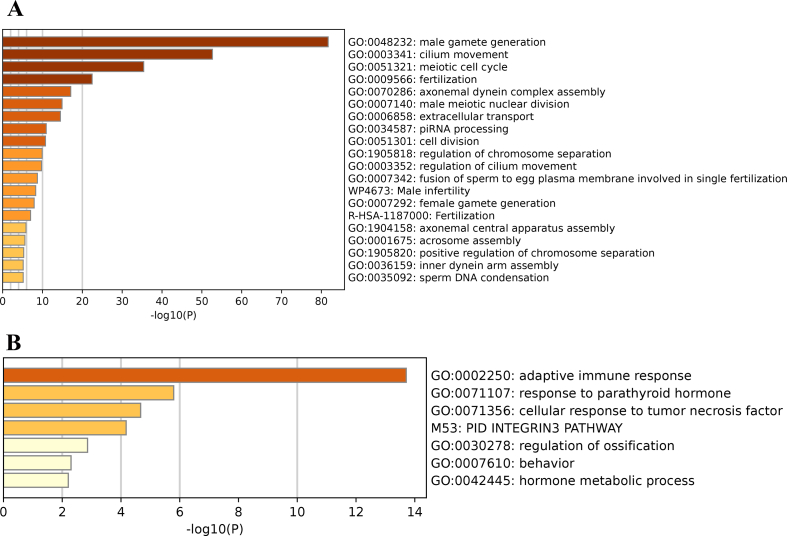


**figs2:**