# Supplementary material for: In situ transcriptomic analysis of spermatocytes in non-obstructive azoospermia reveals senescence-like states in arrested spermatocytes
Source: Genes Dis. 2024 Jan 3;12(1):101205. doi: 10.1016/j.gendis.2024.101205 (PMC11462241; doi:10.1016/j.gendis.2024.101205)
Supplement: Multimedia component 1 [file mmc1.docx]

**
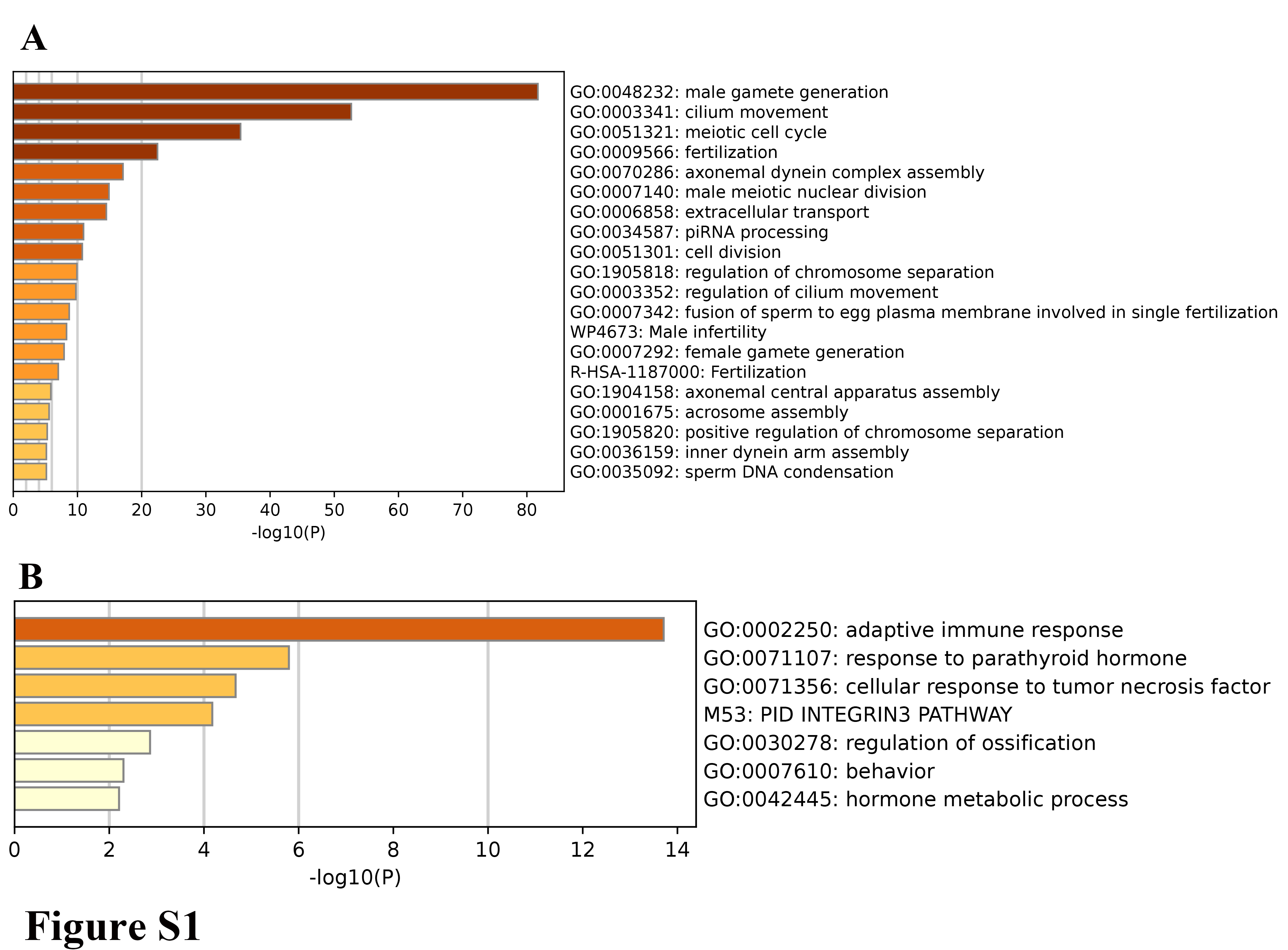
**

**Figure S1**

GO analysis of some expression-alternated genes in SP_NOA group. A) GO analysis of 1739 genes which are completely depressed in SP_NOA group. B) GO analysis of 79 genes which are only expressed in SP_NOA group


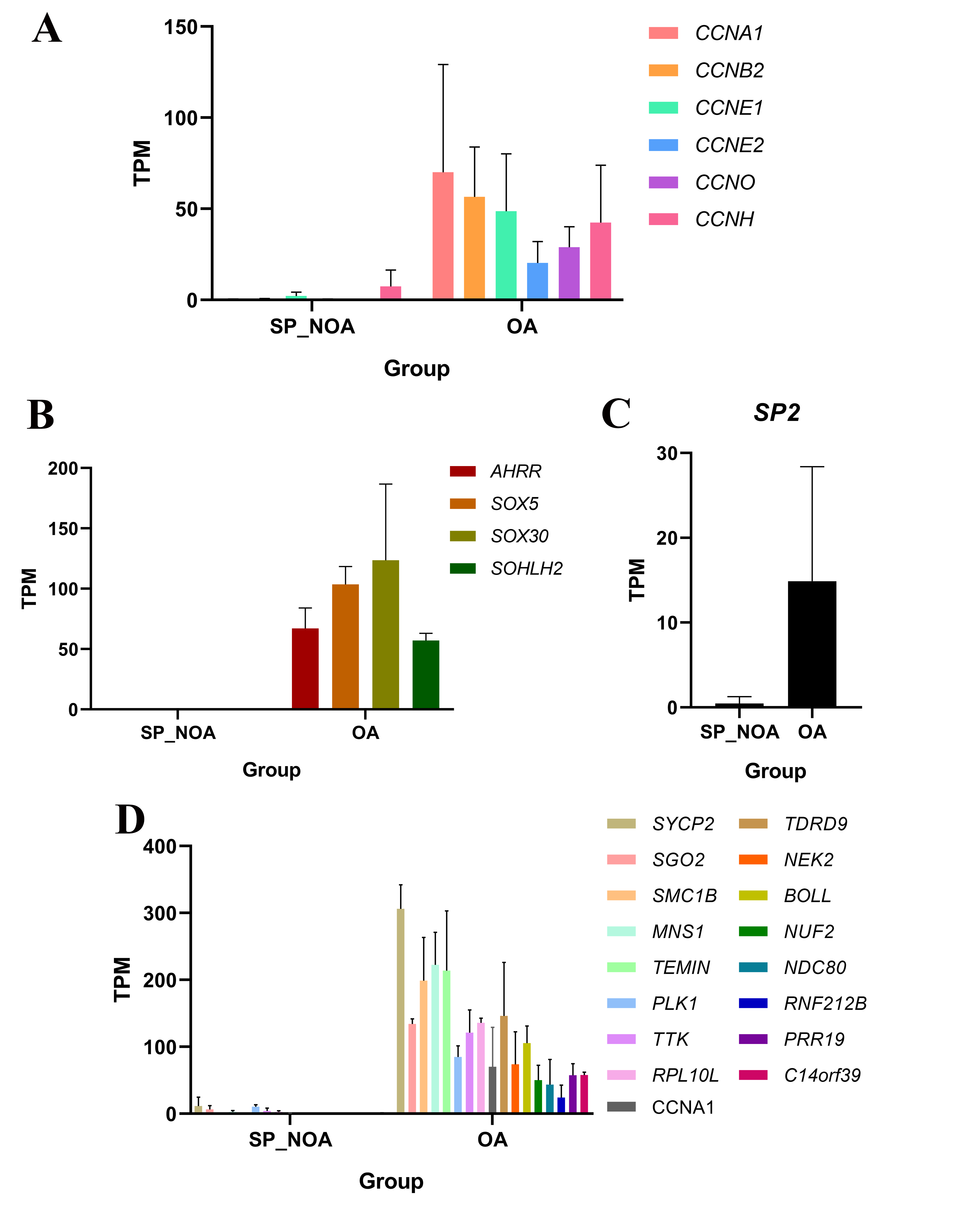


**Figure S2**

TPM value of different functional genes in SP_NOA group and OA group. A) Cyclin family related genes, B) meiotic progression related genes, C) upstream regulator of *RFX5* gene, D) 17 potential target genes relevant to meiosis of *RFX* family regulators
